# A three-dimensional collagen construct to model lipopolysaccharide-induced activation of BV2 microglia

**DOI:** 10.1186/1742-2094-11-134

**Published:** 2014-07-30

**Authors:** Randy Tatt Yhew Haw, Chih Kong Tong, Andrew Yew, Han Chung Lee, James B Phillips, Sharmili Vidyadaran

**Affiliations:** 1 Neuroinflammation Group, Immunology Laboratory, Department of Pathology, Faculty of Medicine & Health Sciences, Universiti Putra Malaysia, 43400 Serdang, Selangor, Malaysia; 2 Genetic & Regenerative Medicine Research Centre (GRMRC) & Department of Obstetrics & Gynaecology, Faculty of Medicine & Health Sciences, Universiti Putra Malaysia, 43400 UPM Serdang, Selangor, Malaysia; 3 Department of Biomaterials & Tissue Engineering, University College London, UCL Eastman Dental Institute, 256 Gray’s Inn Road, London WC1X 8LD, UK

**Keywords:** Microglia, Lipopolysaccharide, Collagen matrix, Three-dimensional cultures

## Abstract

**Background:**

We report a novel method of culturing microglia in three dimension (3D) using collagen as a substrate. By culturing microglia within a matrix, we aim to emulate the physical state of microglia embedded within parenchyma.

**Methods:**

BV2 microglia cell suspensions were prepared with type I collagen and cast into culture plates. To characterise the BV2 microglia cultured in 3D, the cultures were evaluated for their viability, cell morphology and response to lipopolysaccharide (LPS) activation. Conventional monolayer cultures (grown on uncoated and collagen-coated polystyrene) were set up concurrently for comparison.

**Results:**

BV2 microglia in 3D collagen matrices were viable at 48 hrs of culture and exhibit a ramified morphology with multiplanar cytoplasmic projections. Following stimulation with 1 μg/ml LPS, microglia cultured in 3D collagen gels increase their expression of nitric oxide (*NO*) and CD40, indicating their capacity to become activated within the matrix. Up to 97.8% of BV2 microglia grown in 3D cultures gained CD40 positivity in response to LPS, compared to approximately 60% of cells grown in a monolayer (*P* < .05). BV2 microglia in 3D collagen gels also showed increased mRNA and protein expression of inflammatory cytokines *IL-6, TNF-α* and the chemoattractant *MCP-1* following LPS stimulation.

**Conclusions:**

In summary, BV2 microglia cultured in 3D collagen hydrogels exhibit multiplanar cytoplasmic projections and undergo a characteristic and robust activation response to LPS. This culture system is accessible to a wide range of analyses and provides a useful new *in vitro* tool for research into microglial activation.

## Background

Microglia are tissue-specific macrophages of the central nervous system (CNS) and derive from primitive haematopoietic progenitors of erythromyeloid origin [1,2]. These mononuclear phagocytes are the resident immune cells of the CNS, along with other subsets of mononuclear phagocytes including meningeal macrophages, choroid plexus macrophages and perivascular macrophages [3]. Microglia in the brain are disseminated throughout the parenchyma and are highly motile. In the healthy mature CNS, microglia exist mainly in a ramified form, continuously traversing the CNS and using their cytoplasmic processes to sample the tissue environment [4].

Although microglia have long been thought of as merely a stromal cell, microglia research has now revealed crucial roles for these cells in inflammation and in different stages of neurodevelopment. Homeostatic changes in the CNS rapidly trigger a reactive form of microglia, characterised by a shift to amoeboidal morphology, increased motility, proliferation and release of inflammatory mediators [5]. In the embryonic brain, microglia associate closely with apoptotic cells, presumably to promote developmental neuron death and phagocytose the ensuing cellular debris [6]. During postnatal development, microglia play a role in synaptic pruning by engulfing synaptic material [7,8]. In adult hippocampal neurogenesis, microglia provide an important housekeeping role by phagocytosing and clearing apoptotic newborn neurons [9]. These functions of the microglia have led to the recognition of the significance of this cell in brain research.

Homogenous cell cultures are a valuable approach for neuroscience research that allows monitoring of the cell population of interest in a carefully controlled environment. Monolayer cultures fail to recreate the 3D spatial arrangement of cells and matrices present in tissues, and stiff plastic substrates do not resemble the physical environment of the CNS. Culture systems that better mimic the behaviour of microglia within a 3D milieu would allow scrutiny of these cells in a more relevant tissue-like microenvironment. Brain slice culture allows *in situ* examination of cellular responses, and has been used to study microglial responses to carcinomas [10] and cerebral amyloidosis [11]. However, downstream analysis of individual cell populations can be complicated and the local cellular environment is complex.

Here, we describe a 3D culture system utilising type I collagen as the basal substrate to provide a matrix for the culture of microglial cells. Type I collagen is a matrix material that is easily manipulated, is widely used in culture models [12], and has previously been used to develop 3D cultures for astrocytes and neurons [13-15]. Being a simple matrix, it also serves as a suitable baseline scaffold on which the deposition of other extracellular matrix (ECM) molecules can be detected. With control over the seeding density and the chemical environment within the gels, we are able to examine specific features of microglia in a 3D matrix with ease of monitoring the cells compared to *in vivo* models or brain slice cultures.

In the laboratory we routinely culture BV2 microglia, a cell line of murine origin immortalised with v-raf/v-myc oncogenes and commonly used in microglia studies. The BV2 microglia are similar in morphology to isolated microglia, express inflammatory mediators and display phagocytic activity [16]. To stimulate the BV2 microglia into an inflammatory phenotype, the bacterial cell wall component lipopolysaccharide (LPS) is used. By examining morphology, viability and activation status (by evaluating nitric oxide production along with CD40 and inflammatory cytokine expression) of BV2 microglia in 3D constructs and comparing them to conventional monolayer cultures, we characterise microglia cultured in 3D and report a model for microglial activation in a 3D collagen matrix using LPS.

## Methods

### BV2 cells

BV2, an immortalised mouse microglia cell line, was cultured in high glucose Dulbecco modified Eagle medium (DMEM; Gibco, Carlsbad USA) supplemented with 5% foetal bovine serum (Gibco, Carlsbad USA), 6.25 μg/ml insulin (Sigma-Aldrich, St. Louis USA), 1X non essential amino acid (Gibco, Carlsbad USA), 1% penicillin and streptomycin ( i-DNA, Singapore), 0.5% fungizone (Gibco, Carlsbad USA) and 0.1% gentamicin (Gibco, Carlsbad USA). The cultures were maintained in a humidified incubator at 37°C with 5% CO^2^:95% air.

### Culture of BV2 cells on monolayer surfaces and in three-dimensional collagen gels

BV2 cells were harvested at 70-90% confluency and counted using a haemocytometer. Cultures up to passage 20 were used for experiments. The seeding density for all culture conditions and downstream assays was 0.3 × 10^6^ cells/well. For uncoated and collagen-coated monolayer cultures, cells were seeded in 6-well plates. For coated monolayer cultures, culture plates were precoated with type I rat tail collagen (First Link UK Ltd, Birmingham, UK) in 0.6% acetic acid for 30 min. The culture flasks were then rinsed with supplemented DMEM to remove any traces of acid.

For 3D cultures, collagen gels were prepared by adding 10% v/v cell suspension in supplemented DMEM, 10% v/v 10X minimum essential medium (MEM; Sigma-Aldrich St. Louis USA) and 80% v/v type I rat tail collagen (2 mg/ml in 0.6% acetic acid; First Link, Birmingham UK). The MEM and collagen were mixed and neutralised using sodium hydroxide as assessed by colour change of the phenol red indicator. Upon neutralisation, the collagen-MEM mixture was gently mixed with the BV2 cell suspension and transferred to culture plates (0.3 ml/well in 24-well plates and 1.2 ml/well in 6-well plates or 35 mm WillCo (WillCo Wells, Amsterdam, Netherlands) dishes; resulting gels were approximately 2-mm thick). The gels were allowed to set in a humidified incubator at 37°C with 5% CO^2^:95% air for 5 to 10 min and subsequently covered with 2 ml supplemented DMEM. The cultures were maintained for up to 48 hrs and cells were retrieved for analysis using 0.25% trypsin for monolayer cultures and 0.125% type I collagenase for 3D cultures.

To stimulate the cultures, BV2 microglia were treated with 1 μg/ml lipopolysaccharide (LPS; *E. coli* serotype O26:B6; Sigma-Aldrich St. Louis USA, Cat. No. L2762) in supplemented DMEM. Controls were subjected to media change only.

### Scanning electron microscopy

Collagen gels with and without BV2 cells were processed based on a modified version of Lizárraga and colleagues [17]. Briefly, samples were prefixed in 2.5% v/v glutaraldehyde at 4°C for 4 hrs and washed with 0.1 M sodium cacodylate buffer. After overnight incubation, samples were post-fixated in 1% osmium tetraoxide. Samples were then dehydrated using a graded series of ethanol followed by immersion in 100% acetone. The samples were then transferred to a Baltec CPD 030 Critical Point Dryer for critical point drying. Samples were coated with gold-palladium in a Baltec SCD 005 Sputter Coater and examined under JEOL JSM-6400 SEM.

### DAPI/propidium iodide staining

BV2 cell viability in all culture conditions was assessed using propidium iodide (PI) and a 4',6-diamidino-2-phenylindole, dihydrochloride (DAPI) counterstain. In brief, 20 μg/ml PI (Molecular Probes, Oregon USA) was added to cultures at 24 and 48 hrs and incubated for 10 min at 37°C. Supernatant was then removed and the cultures rinsed in 1× PBS thrice for 5 min to remove PI residue. Cells were fixed with 4% paraformaldehyde (PFA) at 4°C for 1 hr. The cultures were then incubated with 1 μg/ml DAPI (Molecular Probes, Oregon USA) in 1X PBS with 0.1% Triton-X for 10 min. For the positive control, Triton-X (0.2% in basic DMEM) was used to treat the cells for 5 min prior to PI staining.

### Lactate dehydrogenase assay

Lactate dehydrogenase (LDH) assay is a colourimetric assay that specifically detects the enzyme lactate dehydrogenase. This enzyme is particularly stable and is present in the culture supernatant when cells are damaged. For this assay, three controls were established, namely, the background control (only media), low control (untreated cells) and positive control (cells treated with lysis solution). All cultures were supplied with an equal volume (1.5 ml) of culture medium. Following overnight incubation at 37°C and 5% CO^2^:95% air, cells were treated with 1 μg/ml LPS in phenol red-free, supplemented DMEM or subjected to a media change for untreated cells. At 48 hrs, lysis solution obtained from the Cytotoxicity LDH kit (Roche, Mannheim Germany) was added into the positive control wells. Next, 500 μl of media from each well was aliquoted into tubes and centrifuged at 1,500 rpm for 5 min to remove any debris. Next, 100 μl of the supernatant was transferred into a 96-well plate in triplicate. A reaction mixture was prepared by mixing the catalyst solution and dye solution from the kit and 100 μl of the reaction mixture was added into each well. The 96-well plate was incubated for 20 min prior to measuring the absorbance at a 490-nm wavelength using a microplate reader (Dynex, Virginia United States). Readings from background controls were subtracted from all reaction absorbance readings.

### Lectin staining

BV2 cell morphology was assessed using lectin histochemical staining. Briefly, cells were fixed with 4% paraformaldehyde (PFA) in 1X PBS for 1 hr followed by permeabilisation with 0.2% Triton-X in 1× PBS for 30 min. Cells were then incubated with fluorescein isothiocyanate (FITC)-conjugated tomato lectin (1:300 dilution in 0.2% of Triton-X in 1X PBS; Sigma-Aldrich, St Louis USA) for 1 hr. Nuclei were counterstained with 0.1 μg/ml DAPI for 5 min before cells were viewed and photographed with an inverted fluorescence microscope (Olympus, Tokyo Japan) and laser scanning confocal microscope (Leica DMIL, Wetzlar Germany).

### CD40 immunophenotyping

For immunophenotyping, cells were harvested from cultures and resuspended in 100 μl of 1× PBS. They were then incubated with Fixable Viability Dye eFluor™ 780 (eBioscience, San Diego USA) at 1:1000 dilution for 15 min, followed by an anti-mouse CD40-FITC antibody (1:100 dilution; BD Pharmigen, San Diego USA) for 30 min at 4°C. Cells were then washed and resuspended in 1× PBS before being analysed by a FACS Fortessa Cytometer (BD Biosciences, San Jose, CA USA). Gating was applied to identify intact cells according to forward and side scatter plots; dead cells were excluded from analysis using the Fixable Viability Dye eFluor™ 780 staining. Data were analysed using the FACS Diva software.

### Griess assay

This assay was performed to study the activation of BV2 cells in a resting state and LPS-treated state by determining the production of nitric oxide (NO) at 36 and 48 hrs, respectively. BV2 cells were seeded in 6-well plates for monolayer cultures and 24-well plates for 3D collagen cultures at a seeding density of 0.3 × 10^6^ cells/well. All cultures were supplied with an equal volume (2 ml) of culture medium. Following overnight incubation at 37°C and 5% CO_2_, cells were treated with 1 μg/ml LPS in phenol red-free, supplemented DMEM. For the Griess assay, 200 μl of media from each well was aliquoted into tubes and centrifuged at 1,500 rpm for 5 min to remove any debris. Next, 50 μl of the supernatant was transferred into a 96-well plate in triplicate. A series of sodium nitrite (NaNO_2_) standards for the Griess assay were prepared by serial dilution, ranging from 0 μM to 100 μM. Griess reagent was freshly prepared by dissolving sulphanilamide (Sigma-Aldrich, United States) and N-(1Naphtyl)ethylenediamine (Sigma-Aldrich, St Louis United States) with phosphoric acid. Fifty microliters of Griess reagent was added to each well before absorbances were measured with a microplate spectrometer (Dynex, Virginia United States) at 530 nm wavelength. The NO_2_^-^ concentration was then evaluated by normalising the absorbance reading using the graph equation obtained from the plotted standard graph.

### Detection of cytokine expression with reverse transcriptase quantitative PCR and a cytokine bead array

The mRNA expression of *MCP-1, IL-6, IL-1β, IL-12β* and *TNF-α* of BV2 microglia was assessed with reverse-transcriptase quantitative PCR (RT-qPCR). The RNA of BV2 microglia was isolated using the RNeasy Plus Mini Kit (Qiagen, Limburg Germany) after a 6-hr stimulation with LPS. The isolation process was conducted according to the kit’s manual. The yield of total RNA was quantified by optical density (OD) readings at 260 nm, and the purity was estimated by the 260:280 nm ratio. Using the SuperScript III Reverse Transcriptase Kit (Invitrogen, Carlsbad USA), an equivalent amount of RNA samples (500 ng) were then reverse transcribed into single stranded cDNA in a reaction mixture consisting of 0.5 mM dNTP mix, 2.5uM oligo d(T)_20_, 0.01 M DTT, 40U RNAseOUT™, 1× RT buffer and 200U Superscript™ RT prepared in 20uL per reaction, according to the manufacturer’s protocol. The primers and housekeeping genes (*Hmbs, Pgk1* and *Psmb2)* were designed and probes selected using ProbeFinder Version 2.49 (Universal Probe Library Assay (UPL) Design Center, Roche). Details on the primer sequences are in Additional file 1. PCR was then performed on a LightCycler 480 System (Roche, Basel Switzerland).Conditions for the RT-qPCR were a predenaturing step of 95°C for 10 min; 45 cycles of 95°C for 10 sec, 60°C for 30 sec, and 72°C for 1 sec; and finishing with a cooling step at 40°C for 30 sec [18]. A PCR efficiency of between 90 and 110% and an R-squared value >0.98 were used to define successful assays. Relative quantification of target gene expression in our samples was carried out using the comparative Ct method. We performed intra-sample data normalisation against the three endogenous control reference genes as mentioned above.

For the bead array, culture supernatants were assessed at 48 hrs for expression *of IL-6, IL-10, MCP-1, IFN-γ, TNF,* and *IL-12p70* using the multiplex bead array kit (BD Cytometric Bead Array mouse inflammation kit, BD Biosciences, San Jose, CA, USA). The samples were assayed according to the manufacturer’s instructions with the FACS Fortessa flow cytometer (BD Bioscience, San Jose, CA, USA). The resulting data were analysed with FCAP array software (BD Bioscience, San Jose, CA, USA). Expression of the cytokines was evaluated by determining their respective concentration (pictograms per millilitre) using individual standard curves.

### Statistical analysis

Significance was assessed using GraphPad Prism version 6 (GraphPad Software, CA, USA, http://www.graphpad.com/).

## Results

### Scanning electron microscopy of BV2 microglia and collagen gel structure

**Figure 1:**
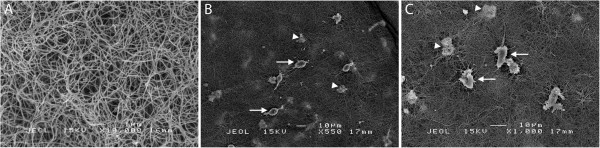
**Scanning electron micrographs of three-dimensional (3D) collagen gel and microglia cultured in 3D. (A)** Structure and organisation of fibrils of 2 mg/ml collagen gels. **(B,C)** Low magnification images show microglia on the surface (arrows) and embedded (arrowheads) within the collagen gels. Magnification as indicated on each micrograph.

Examination of the collagen gel structure was performed using scanning electron microscopy. The matrix consists of a dense fibrillar network, with numerous interfibrillar spaces (Figure 1A). Microglia were detected on the surface of the gels and also beneath the surface (arrows and arrowheads in Figure 1B,C).

### Morphology and viability of BV2 microglia cultured in monolayer and three dimensions

**Figure 2:**
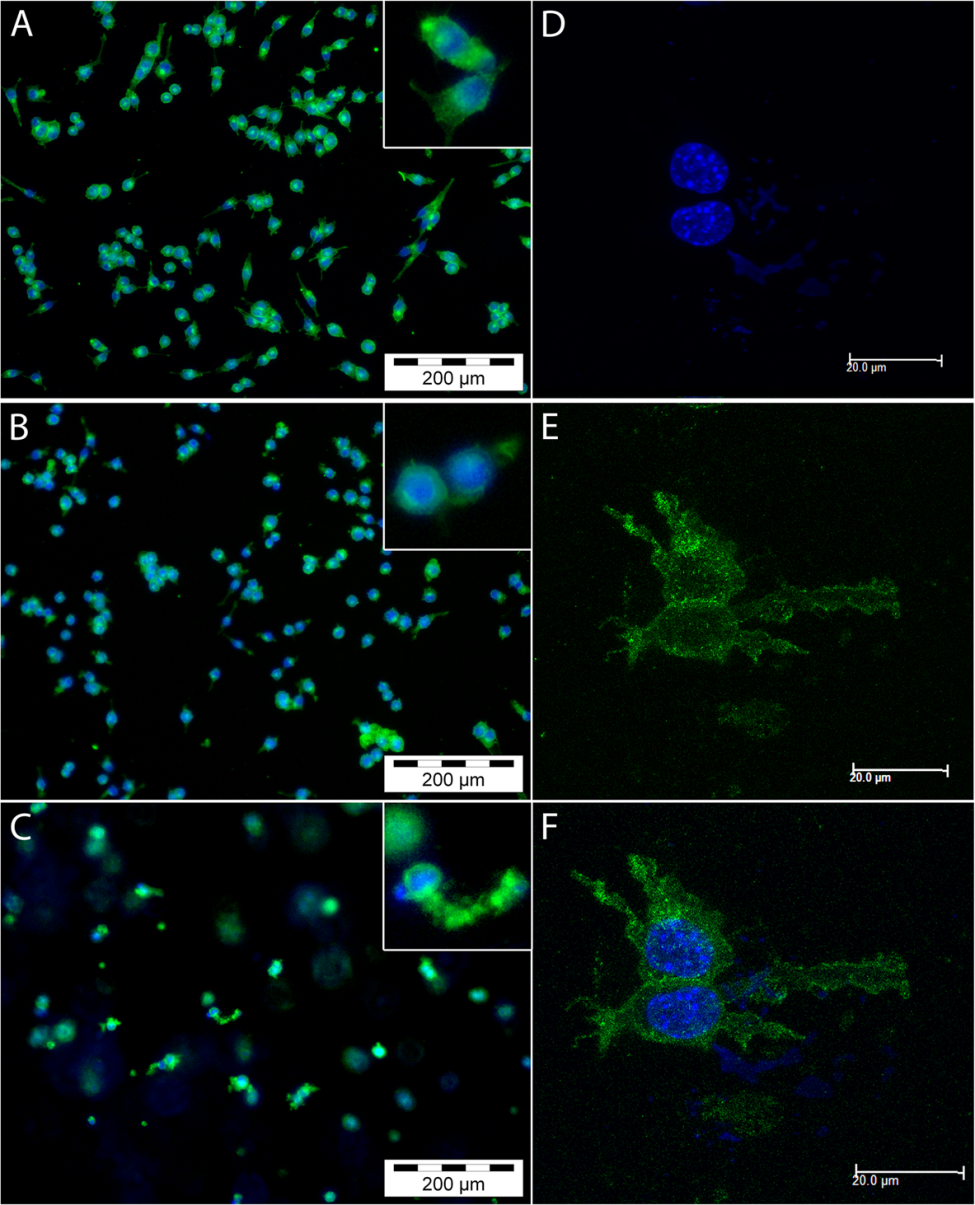
**Morphology of microglia cultured in monolayer and three-dimensional (3D) systems.** Microglia cultured in monolayer **(A)**, coated monolayer **(B)** and 3D collagen gels **(C,D,E,F)** were stained with fluorescein isothiocyanate (FITC)-tagged lectin and 4',6-diamidino-2-phenylindole, dihydrochloride (DAPI) and viewed with fluorescent **(A-C)** or confocal **(D-F)** microscopy.

**Figure 3:**
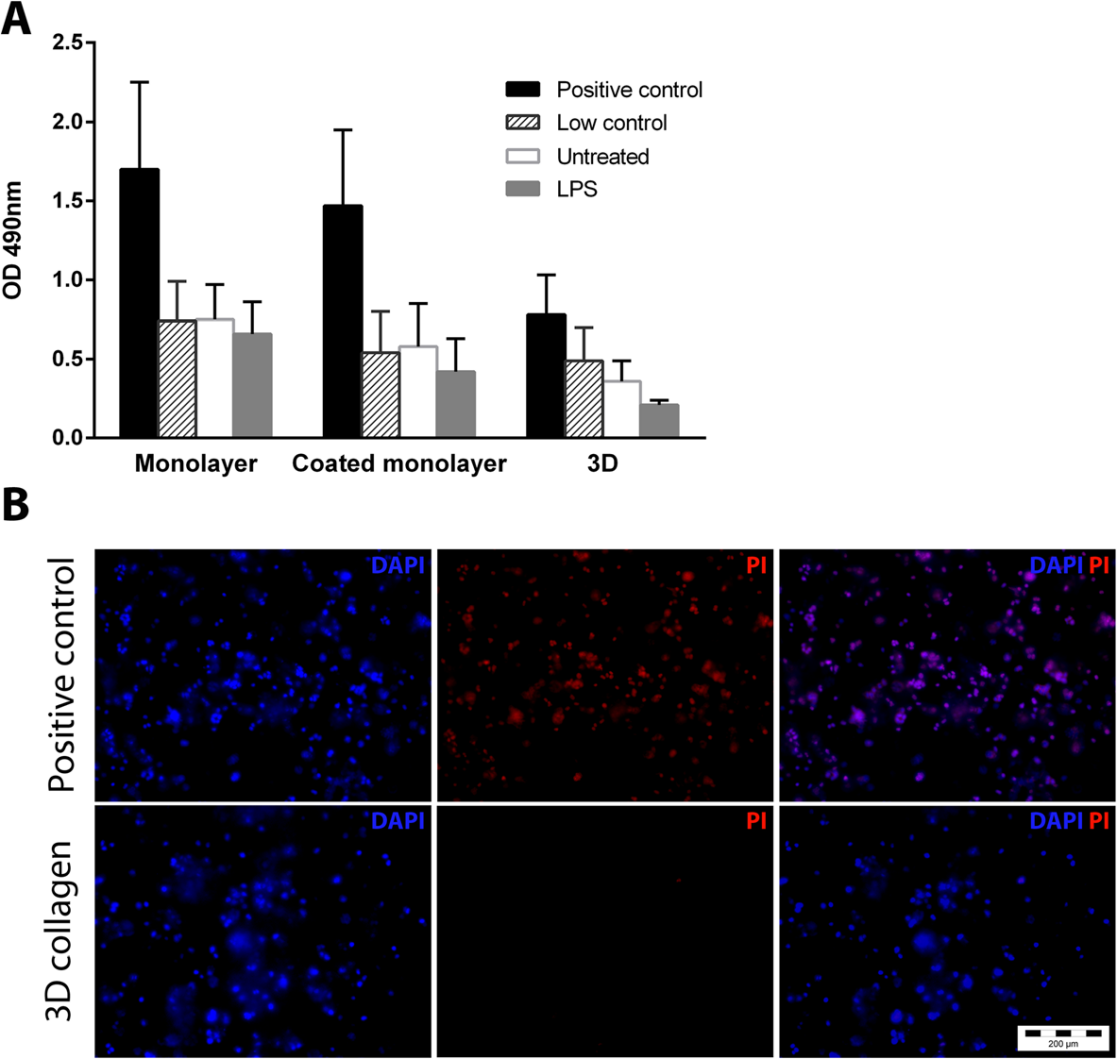
**BV2 microglia are viable in three-dimensional (3D) collagen gels. (A)** BV2 microglia in monolayer, coated monolayer and 3D culture conditions were assessed for lactate dehydrogenase (LDH) activity at 48 hrs. Data are mean ± SD from three independent experiments. **(B)** Cells were stained with 4',6-diamidino-2-phenylindole, dihydrochloride (DAPI) and propidium iodide (PI) to assess viability at 48 hrs post-culture. LPS, lipopolysaccharide.

To examine the morphology of microglia in the various culture systems, we performed staining of cells with FITC-tagged lectin. Microglia cultured on uncoated monolayer surfaces mostly exhibited round cytoplasm with some bipolar projections (Figure 2A). In collagen-coated monolayer cultures, the extent of amoeboidal morphology seemed increased (Figure 2B) with cytoplasmic area appearing minimal, indicating the extent of deramification. The morphology of cells cultured in 3D collagen was distinct from monolayer, with clear multiplanar projections (Figure 2C). The microglia were suspended within the collagen matrix and evenly distributed across the width of the gel. When viewed with confocal microscopy, the extent of ramification of microglia cultured in the 3D matrix was evident, with cells displaying long and multidirectional cellular projections (Figure 2F and video in Additional file 2).To determine whether microglia cultured in 3D were viable, the lactate dehydrogenase (LDH) assay and DAPI/PI staining were performed. Compared to the respective positive controls, BV2 microglia in all three culture formats showed low LDH release (Figure 3A). Even following treatment with 1 μg/ml LPS, LDH levels in the culture supernatants remained low. Additionally, BV2 microglia showed negligible PI staining at 24 (data not shown) and 48 hrs post-culture in 3D collagen (Figure 3B).

### CD40 expression of microglia cultured in the three-dimensional collagen matrix

We routinely assess expression of the co-stimulatory molecule CD40 as a measure of the activation status for microglia. Upon exposure to lipopolysaccharide (LPS), microglia secrete inflammatory mediators and upregulate expression of major histocompatibility complex (MHC) class II receptors and CD40 to facilitate antigen presentation to T lymphocytes [19]. Due to these effects, LPS is a common stimulus for activating microglia *in vitro*[20-22], including for our previous work [23-25].

**Figure 4:**
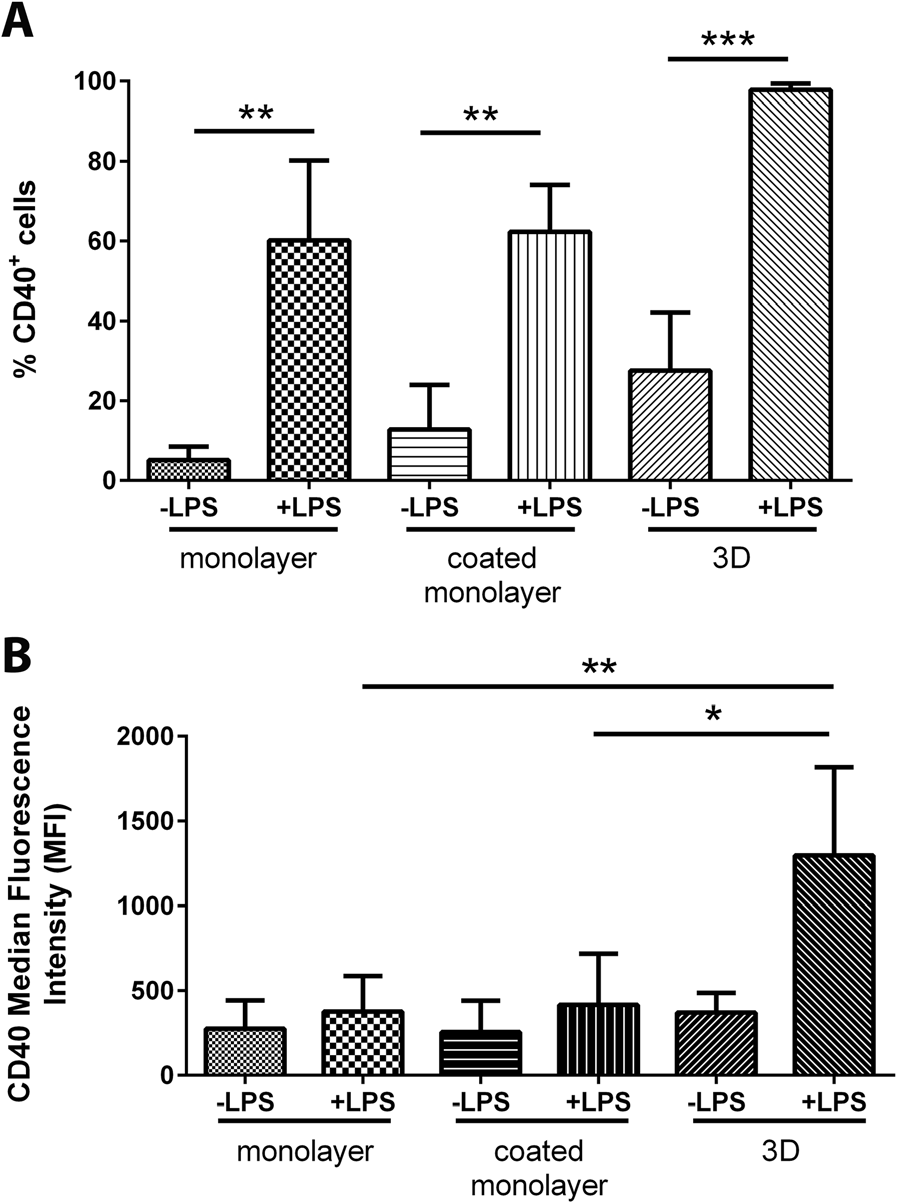
**BV2 microglia in three-dimensional (3D) collagen gels shift as a population to express CD40 in response to lipopolysaccharide (LPS) stimulation.** BV2 microglia in monolayer, coated monolayer and 3D culture conditions were activated with 1 μg/ml LPS (+LPS) and CD40 expression analysed with flow cytometry at 24 hrs. **(A)** Histograms show percentage of CD40^+^ cells. Data are mean ± SD from three independent experiments. **P < .01, ****P < .001; one-tailed Mann-Whitney U test. **(B)** Histograms show median fluorescence intensity (MFI) of CD40^+^. Data are mean ± SD from three independent experiments. *P < .05, **P < .01 ; Kruskal Wallis with Dunn’s Multiple Comparison test.

The number of microglia expressing basal CD40 in 3D cultures was higher (27.6 ± 14.6%) compared to cells grown in uncoated monolayer cultures (5.1 ± 3.39%) (*P* < .05; Kruskal-Wallis with Dunn’s multiple comparison test). When stimulated with LPS, the number of CD40^+^ BV2 microglia increased by 70% (*P* < .001), with almost the entire population of BV2 cells cultured in 3D collagen shifting to a CD40^+^ phenotype at 24 hrs (97.8 ± 1.5%; Figure 4A). CD40^+^ BV2 cells in uncoated and collagen-coated monolayer cultures also increased from 5.1 ± 3.4% and 12.8 ± 11.2% to 60.2 ± 20.0% and 62.3 ± 11.8%, respectively (Figure 4A; *P* < .05). The increase in CD40^+^ cells was significantly higher for 3D cultures compared to uncoated and collagen-coated monolayer cultures (*P* < .05; Kruskal-Wallis with Dunn’s multiple comparison test). Achieving a homogenous population shift may indicate a uniform level of activation amongst the microglia, an effect that appears to be rendered by the 3D collagen culture.

The median fluorescence intensity (MFI) readouts on a flow cytometer indicate whether cells within a positive population express the marker in question at different intensities. Using MFI, we show that not only do the number of CD40^+^ BV2 microglia increase in 3D culture, but the degree of their CD40 expression also increases 3-fold compared with cells grown on uncoated monolayer and collagen-coated monolayer surfaces (*P* < .05; Figure 4B). Also, microglia in monolayer cultures appear to respond to LPS by acquiring CD40 expression, and not by increasing the level of expression (Figure 4B). Collectively, microglia in 3D culture appear to be activated in a more homogenous fashion, and to a greater extent than cells in monolayer cultures. Interestingly, the intensity of CD40 expression in unstimulated cultures remained similar between all three culture formats. Therefore, although the number of CD40^+^ cells is higher in 3D cultures compared to monolayer, the degree of expression per cell is similar.

### Nitric oxide and inflammatory cytokine expression by microglia cultured in three dimensions

Disease or damage within the CNS triggers production of nitric oxide (NO) by microglia and astrocytes [26]. The inducible nitric oxide synthase (iNOS) isoform of *NO* synthase is responsible for secretion of large, continuous amounts of NO by these glial cells during inflammation, as compared to neuronal NOS (nNOS) that is constitutively expressed in neurons and is believed to have a physiological role in the brain [26].

**Figure 5:**
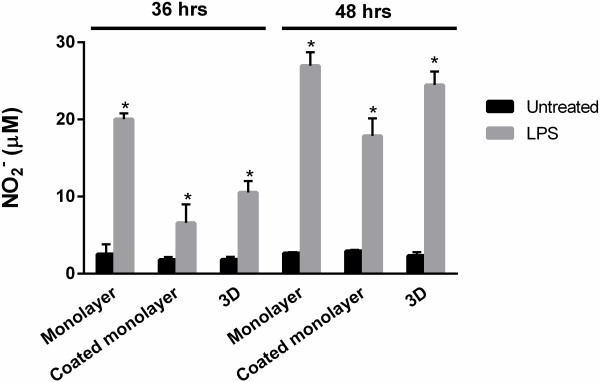
**BV2 microglia cultured in three-dimensional (3D) collagen gels express nitric oxide (NO) in response to lipopolysaccharide (LPS) stimulation.** BV2 microglia in monolayer, coated monolayer and 3D culture conditions were activated with 1 μg/ml LPS and assessed for NO_2_^*-*^ expression at 36 and 48 hrs with the Griess assay. Data are mean ± SD from three independent experiments.

To assess NO production by BV2 microglia in monolayer and 3D cultures, cells were seeded in the different culture formats with equal seeding number per well. Microglia in monolayer and 3D cultures had negligible NO production (<3 μM at both 36 and 48 hrs; Figure 5), indicating that the culture conditions alone do not induce NO expression. Following stimulation with LPS, BV2 microglia in all culture formats showed an induction of *NO* expression, approximately 4 to 8 times higher at 36 hrs and 6 to 10 times higher at 48 hrs compared to untreated BV2 (*P* < .05). Negligible NO induction was detected at 24 hrs post-LPS (data not shown). At 36 hrs, BV2 grown as uncoated monolayer cultures produced the highest amount of NO (20.03 ± 0.76 μM), followed by collagen-coated monolayer cultures (6.59 ± 2.39 μM) and 3D cultures (10.54 ± 1.46 μM). These levels increased for uncoated monolayer, collagen-coated monolayer and 3D culture at the 48-hr time point to 26.96 ± 1.76 μM, 17.87 ± 2.27 μM and 24.47 ± 1.75 μM NO*,* respectively. The NO expression by LPS-treated BV2 cells in 3D cultures was not significantly different compared to microglia cultured in uncoated or collagen-coated monolayer cultures at either time points (*P* > .05; Kruskal-Wallis with Dunn’s multiple comparison test).

**Figure 6:**
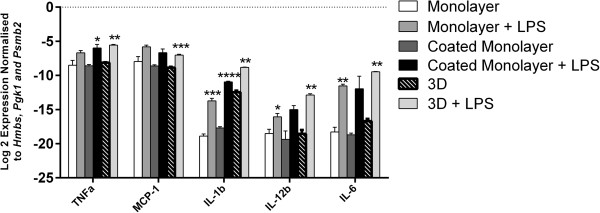
**Reverse-transcriptase quantitative PCR ( RT-qPCR) demonstrates the level of inflammatory cytokines by BV2 microglia 6 hrs after lipopolysaccharide (LPS) stimulation.** The values have been normalised to three housekeeping genes (*Hmbs, Pgk1 and Psmb2). TNF, MCP-1, IL-1b, IL-6* and *IL-12b* were upregulated in stimulated BV2 microglia for three-dimensional (3D) cultures. Data are mean ± SEM from three independent experiments. *P < .05, **P < .01, ***P < .001, ****P < .0001; Welch’s t test*.*

The response of microglia in 3D cultures to LPS was also evaluated by determining expression of inflammatory cytokines mRNA and protein. At 6 hrs, mRNA expression of all inflammatory cytokines tested (*MCP-1, IL-6, IL-1β, IL-12β* and *TNF-α*) was significantly upregulated in BV2 microglia cultured in 3D, relative to housekeeping gene expression (Figure 6 and Additional file 3). Similar to mRNA expression, protein levels for *IL-6, TNF-α* and *MCP-1* were increased following LPS stimulation in 3D cultures. At 48 hrs of LPS stimulation, BV2 microglia in 3D cultures recorded significantly higher expression of *IL-6, TNF-α* and *MCP-1* compared to untreated BV2 cells (*P* < .05; Mann Whitney Test) (Table 1). Levels of *IL-6, TNF-α* and *MCP-1* increased by 1,998.3 pg/ml, 1,735.9 pg/ml and 5,119.0 pg/ml respectively compared to basal (untreated) levels. As we have shown before [23], levels of *IFN-γ, IL-10* and *IL-12p70* were unaffected by LPS stimulation. Additionally it was observed that unstimulated BV2 microglia in 3D cultures demonstrated markedly lower *MCP-1* levels compared to monolayer cultures (*P* < .05; Kruskal-Wallis with Dunn’s multiple comparison test), which was significantly upregulated with LPS stimulation. *MCP-1* expression for LPS-treated BV2 cells in monolayer and coated monolayer cultures was not significantly different from unstimulated cultures.

**Table 1:** **BV2 microglia in three-dimensional (3D) cultures show increased expression of *IL-6, TNF-α *** **and *MCP-1 *** **at 48 hours post-lipopolysaccharide (LPS) stimulation**

	**Untreated**			**LPS-treated**		
	***IL-6***	***TNF-α***	***MCP-1***	***IL-6***	***TNF-α***	***MCP-1***
**Monolayer**	1.7 ± 0.3	252.3 ± 120.5	9 447.0 ± 226.8	2 476.0 ± 833.6**	3 823.0 ± 1189.0**	10 459.0 ± 1, 639.0^ns^
**Coated monolayer**	1.6 ± 0.99	217.6 ± 181.8	9 231.0 ± 2268.0	1 764.0 ± 324.3**	2 357.0 ± 500.9**	10 936.0 ± 1292.0^ns^
**3D**	0.7 ± 0.8	8.1 ± 3.1	284.0 ± 73.5	1 999.0 ± 685.2*	1 744.0 ± 911.6*	5 403.0 ± 517.6*

Values are expressed in pg/ml, and assayed using the Cytometric Bead Array. Data are mean ± SD from five independent experiments. *p < .05, **p < .01; two-tailed Mann-Whitney U test, as compared to untreated controls. ns, not significant.

## Discussion

Conventional monolayer cell cultures involve the growth of cells on a plastic surface, often coated with extracellular matrix proteins such as collagen and laminin to encourage adherence. Several fundamental disparities exist between these conventional monolayer cultures and cells *in situ*, namely cells cultured in monolayer grow flat, may receive cues from the stiff matrix, do not grow in a stratified manner and have only one side adhering to the plastic surface. This also means that perfusion of nutrients for the cells only occurs via the non-adhered side. For highly reactive cells such as the microglia, these culture conditions could affect their behaviour. The BV2 microglia cell line is commonly used for microglia research, is well-characterised and is routinely studied in our laboratory. To approach *in vitro* BV2 microglia cultures in a more relevant manner, we sought to culture BV2 microglia within a collagen matrix to mimic the mechanical relationship of these cells with tissue. We demonstrate that BV2 microglia grown in 3D collagen are viable, embedded and distributed within the matrix, with a ramified morphology. The ability of microglia to grow within collagen matrices also allows for multiplanar projections of the cytoplasm, which would be impossible to achieve with conventional monolayer cultures.

Using lipopolysaccharide (LPS), we developed an activation model for BV2 microglia cultured in 3D. LPS is a bacterial cell wall component that triggers microglia activation via Toll-like receptor 4 [19]. With 1 μg/ml LPS, a dose routinely used to activate microglia in conventional monolayer cultures [20,24,25], BV2 microglia in 3D cultures significantly increased expression of the cell surface receptor CD40, nitric oxide (NO), *IL-6, TNF-α* and *MCP-1*. This demonstrates that both cells and supernatant of 3D collagen cultures can be assayed, demonstrating the suitability of this culture system to accommodate a range of tests. For CD40 expression, microglia cultured in 3D showed a higher number of cells with basal expression of CD40 compared to the monolayer cultures. The NO levels among all three cultures in unstimulated conditions, however, were similar, indicating that collagen alone (in monolayer or 3D formats) does not trigger NO production in unstimulated BV2 cells. The extent of CD40 expression per cell (shown by MFI readings) was also similar between microglia cultured in monolayer and 3D. Importantly, LPS stimulation triggers a uniform upregulation of CD40 in 3D cultures compared to cells cultured in monolayer formats, with the entire population of microglia acquiring CD40 expression. Therefore, it appears that BV2 microglia cultured in 3D acquire CD40 towards LPS in a more homogenous manner. The microglia cultured in 3D also expressed significant amounts of NO in response to LPS.

Beyond the parameters assayed here, the 3D collagen culture model may be appropriate for studying microglial deposition of ECM material as it offers a matrix of simple composition compared to other more complex substrates such as Matrigel™ [14]. With the data now to show that BV2 microglia are activated by LPS within 3D collagen cultures, we are keen to utilise this model for our main research approach of modulating inflammatory responses of microglia. The growth of microglia within an environment that is more physically and spatially relevant than conventional flat plastic culture plates, along with the ability to stimulate the microglia into an activated phenotype, gives us access to a more refined *in vitro* tool for microglia research.

## Conclusions

By culturing microglia within a simple matrix, we offer a more relevant *in vitro* model compared to conventional monolayer cultures where microglia grow flat on a plastic surface. BV2 microglia cultured in 3D collagen constructs render cells that are viable, well distributed within the matrix, ramified in morphology and activated into an inflammatory phenotype following LPS stimulation.

## Abbreviations

CNS: Central nervous system; DAPI: 4',6-diamidino-2-phenylindole, dihydrochloride; ECM: Extracellular matrix; FITC: Fluorescein isothiocyanate; iNOS: inducible nitric oxide synthase; LDH: Lactate dehydrogenase; LPS: Lipopolysaccharide; MFI: Median fluorescence intensity; nNOS: neuronal nitric oxide synthase; NO: Nitric oxide; OD: Optical density; PFA: Paraformaldehyde; PI: Propidium iodide; 3D: three-dimensional.

## Competing interests

The authors declare that they have no competing interests.

## Authors’ contributions

RHTY performed the cell cultures, LDH assay, Griess assay and cytokine bead array. TCK established the 3D collagen cultures, performed the confocal microscopy, immunophenotyping and viability staining. AY performed the scanning electron microscopy. LHC performed the RT-qPCR experiments. SV and JBP conceptualised the study. SV wrote the manuscript. All authors analysed the data and read and approved the final manuscript.

## Supplementary Material

Additional file 1 List of primers and UPL probes used for reverse-transcriptase quantitative PCR (RT-qPCR) validations. Click here for file

Additional file 2 Video demonstrating three-dimensional (3D) image of a BV2 microglia with the 3D collagen gel. Click here for file

Additional file 3 **Reverse-transcriptase quantitative PCR (RT-qPCR) analyses of *TNF, MCP-1, IL-b, IL-12b *** **and *IL-6 *** **mRNA.** Click here for file
