# Supplementary material for: A three-dimensional collagen construct to model lipopolysaccharide-induced activation of BV2 microglia
Source: J Neuroinflammation. 2014 Jul 30;11:134. doi: 10.1186/1742-2094-11-134 (PMC4128540; doi:10.1186/1742-2094-11-134)
Supplement: Additional file 1 — List of primers and UPL probes used for reverse-transcriptase quantitative PCR (RT-qPCR) validations. [file 1742-2094-11-134-S1.pdf]

Additional File 1. List of primers and UPL probes used for RT-qPCR validations.

Gene	Forward primers	GC%	Reverse primers	GC%	Amplicon size (nt)	Ta (^{0}C)	UPL Probe (#)	Remarks
Il1b	tgtaatgaaagacggcacacc	48	tcttctttgggtattgcttgg	43	68	59	78	ISP
Il6	gctaccaaactggatataatcagga	40	ccaggtagctatggtactccagaa	50	78	60	6	ISP
Il12b	ccagcactcaggagaccaag	60	gaatttctgtgtggcactgg	50	99	60	64	ISP
Mcp1	catccacgtgttggctca	56	gctgctggtgatcctcttg	58	118	60	19	ISP
Tnf	tcttctcattcctgcttgtgg	48	ggtctgggccatagaactga	55	128	59	49	ISP
Pgk1	tacctgctggctggatgg	61	cacagcctcggcatatttct	50	65	60	108	HKG
Psmb2	gagggcagtggagcttctta	55	aggtgggcagattcaagatg	50	71	60	25	HKG
Hmbs	aaagttccccaacctggaat	45	ccaggacaatggcactgaat	50	98	60	42	HKG

Ta = Annealing temperature; ISP = Intron-spanning primers; NISP = Non intron-spanning primers; HKG = Housekeeping genes
