# Supplementary material for: A three-dimensional collagen construct to model lipopolysaccharide-induced activation of BV2 microglia
Source: J Neuroinflammation. 2014 Jul 30;11:134. doi: 10.1186/1742-2094-11-134 (PMC4128540; doi:10.1186/1742-2094-11-134)
Supplement: Additional file 3 — Reverse-transcriptase quantitative PCR (RT-qPCR) analyses of TNF, MCP-1, IL-b, IL-12b and IL-6 mRNA. [file 1742-2094-11-134-S3.pdf]

Additional File 3. RT-qPCR analysis of TNF, MCP-1, IL-b, IL-12b and IL-6 mRNA.

Description	Tnf	Mcp1	Il1b	Il12b	Il6
Log2 Normalised Mean Expression Value for Monolayer	-8.51051	-7.9588	-18.8985	-18.5132	-18.2825
Log2 Normalised Mean Expression Value for Monolayer + LPS	-6.69269	-5.80934	-13.7278	-16.0642	-11.5454
Log2 Normalised Mean Expression Value for Coated Monolayer	-8.59589	-8.61273	-17.6926	-19.3737	-18.6941
Log2 Normalised Mean Expression Value for Coated Monolayer + LPS	-5.98464	-6.66907	-10.9499	-14.9993	-11.9468
Log2 Normalised Mean Expression Value for 3D	-8.14004	-8.87032	-12.4523	-18.5585	-16.6984
Log2 Normalised Mean Expression Value for 3D + LPS	-5.57485	-7.02454	-8.83086	-12.8727	-9.4628
Standard Error of Mean for Monolayer	0.709175	0.730395	0.324047	0.633583	0.692027
Standard Error of Mean for Monolayer + LPS	0.325786	0.250015	0.373621	0.4923	0.249844
Standard Error of Mean for Coated Monolayer	0.196812	0.163058	0.184242	1.228887	0.234416
Standard Error of Mean for Coated Monolayer + LPS	0.518347	0.536352	0.109437	0.586572	1.819462
Standard Error of Mean for 3D	0.073256	0.137194	0.220161	0.559362	0.324501
Standard Error of Mean for 3D + LPS	0.107998	0.123394	0.03888	0.177059	0.024401
P-value of Monolayer / Monolayer + LPS	0.0001	0.0006	0.0029	0.0056	0.0019
P-value of Coated Monolayer / Coated Monolayer + LPS	0.0255	0.058	< 0.0001	0.0521	0.0634
P-value of 3D / 3D + LPS	0.1082	0.086	0.0005	0.041	0.0054
